# A Novel Catecholopyrimidine Based Small Molecule PDE4B Inhibitor Suppresses Inflammatory Cytokines in Atopic Mice

**DOI:** 10.3389/fphar.2018.00485

**Published:** 2018-05-11

**Authors:** Baskaran Purushothaman, Parthasarathy Arumugam, Joon Myong Song

**Affiliations:** College of Pharmacy, Seoul National University, Seoul, South Korea

**Keywords:** phosphodiesterase-4B, catecholopyrimidine, cyclic adenosine mono phosphate, T-helper cells, mast cells

## Abstract

Degradation of cyclic adenosine mono phosphate (cAMP) by phosphodiesterase-4B (PDE-4B) in the inflammatory cells leads to elevated expression of inflammatory cytokines in inflammatory cells. Suppression of cytokines has proved to be beneficial in the treatment of atopic dermatitis (AD). Henceforth, application of PDE4B specific inhibitor to minimize the degradation of cAMP can yield better results in the treatment of AD. PDE4B specific inhibitor with a limited side effect is highly warranted. Herein, we synthesized a novel PDE4 inhibitor, compound **2** comprising catecholopyrimidine core functionalized with trifluoromethyl (-CF_3_) group. PDE4B inhibitory potential and specificity of novel compounds were evaluated by PDE inhibitor assay. *In vivo* efficacy of the compounds was analyzed using DNCB-induced NC/Nga mice. IgE, CD4+ T-helper cell infiltration, and cytokine profiles were analyzed by ELISA and immunohistochemistry techniques. Toluidine blue staining was performed for mast cell count. PDE4 inhibitor assay confirmed that compound **2** specifically inhibits PDE4B. *In vivo* analysis with DNCB-induced NC/Nga mice confirmed that compound **2** suppressed the levels of pro-inflammatory cytokines such as TNF-α, IL-4, IL-5, and IL-17. Furthermore, compound **2** significantly reduced the infiltrative CD4+ T-helper cells, mast cells and IgE levels in atopic tissue. The *in vitro* and *in vivo* data suggested that compound **2** specifically inhibit the PDE4B and the symptoms of the AD in atopic mice. Compound **2** might constitute a good candidate molecule for the treatment of AD.

## Introduction

Atopic dermatitis (AD) is an inflammatory skin disease that particularly affects young children (Beasley, 1998; Odhiambo et al., 2009). Unbearable itching is the major symptom of the AD. It leads to the psychological difficulties such as anxiety, anger, and depression (Simpson et al., 2016). Disruption of epidermal structure and infiltration of abnormal levels of T-helper cells, neutrophils, and mast cells into the dermis are the major pathophysiology of the AD (Wu et al., 2015). Activated lymphocytes at the dermis region constitutively secrete abnormal levels of inflammatory cytokines and contribute to the inflammation of the skin (Boguniewicz and Leung, 2011). Topical application of corticosteroids is commonly prescribed to treat AD. However, long-term administration of corticosteroids is required to treat AD, it eventually leads to the systemic side effects and cutaneous atrophy. Hence, alternative therapy is required to minimize the treatment duration and side effects.

Cyclic adenosine monophosphate (cAMP) regulates numerous metabolic reactions. The regulatory function of the cAMP is generally cell dependent. cAMP-mediated protein kinase-A (PKA) signaling negatively regulates the expression of pro-inflammatory cytokines in lymphocytes and macrophages (Jimenez et al., 2001; Mosenden and Taskén, 2011). Elevated intracellular levels of cAMP have been implicated in the reduction of lymphocyte-derived cytokine and chemokines. However, phosphodiesterases (PDEs) present in the inflammatory cells diminishes the intracellular levels of cAMP through the hydrolysis of cyclic nucleotides (Arp et al., 2003; Asirvatham et al., 2004; Small-Howard et al., 2005). The PDEs are organized into 11 families based on biochemical properties and sequence homogeneity. The PDE4 enzyme family is mainly involved in the degradation of cAMP and is predominantly expressed in the inflammatory cells such as lymphocytes, neutrophils, and eosinophils. They are subdivided into four subtypes namely PDE4-A, B, C, and D. Recently, PDE4 inhibition has become an attractive alternative therapy for inflammatory diseases. Several oral PDE4 inhibitors like roflumilast are in clinical trials. However, oral administration of PDE4 inhibitors generally induces the central nervous system to stimulate nausea and emesis (Calverley et al., 2007; Konrad et al., 2015). Inhibition of PDE4D isoform in the brain is considered responsible for those systemic effects. Conversely, PDE4B specific PDE inhibitors can alleviate these abnormalities. In addition, topical application of PDE4B inhibitor is expected to circumvent the side effects caused by the oral application. Several PDE4 inhibitors including apremilast are in the clinical trials for AD therapy (Felding et al., 2014; Bissonnette et al., 2016). However, an effective selective PDE4B inhibitor is still required to treat allergic inflammatory disorders of the skin.

Based on the previously reported PDE4 inhibitors, for the first time pyrimidine core containing novel PDE4 inhibitors, compounds **1** and **2** were designed and synthesized in our laboratory (Purushothaman et al., 2017). The compounds **1** and **2** possess a pyrimidine nucleus functionalized with 3,4-dimethoxyphenyl (catechol) scaffold with different functional groups. In compound **1**, 3,4-dimethoxyphenyl group substituted at the 5th position, and morpholine at the 4th position of the pyrimidine nucleus. In compound **2**, pyridyl group was coupled at 5th position and the 3,4-dimethoxyphenyl group was substituted at 4th position of pyrimidine nucleus. The morpholine and catecholopyrimidine core present in the synthesized compounds mimics the nucleotide part of cAMP, which may increase its binding affinity toward PDE4B. In the present study, compound-**1**, and compound-**2** were screened for PDE4B inhibitory potential. Their pharmacological profiles were investigated in detail using *in vitro* and *in vivo* models. This report is mainly focused on the catecholopyrimidine compound **2** since it selectively inhibits the PDE4B at nano-molar levels. The anti-pruritic and anti- cytokine profile of the compound **2** were investigated by using DNCB-induced atopic Nc/Nga mice. Our results confirmed that compound **2** effectively inhibits PDE4B activity, thereby alleviating AD-like symptoms in DNCB-induced NC/Nga mice.

## Materials and Methods

### Synthesis of Compounds 1 and 2

The synthesis and characterization of compounds **1** and **2** are provided in Supplementary Material.

### Phosphodiesterase Enzyme Assay

The enzyme inhibition assay was performed against human PDE enzymes (PDE1A, PDE3A, PDE4A, PDE4B, PDE4C, PDE4D, and PDE7A; BPS Biosciences, San Diego, CA, United States) according to the manufacturer’s instructions (LANCE Ultra cAMP assay kit; Perkin Elmer, United States). In each well, 5 μl of 3 nM cAMP, 2.5 μl of PDE enzyme (0.1 ng/well), and 2.5 μl of inhibitor solution were added, and incubated at 37°C for 1 h. After incubation, 5 μL each of Eu-cAMP and ULight-anti-cAMP detection reagent supplemented with 1 mM of IBMX were added. The reaction mixture was incubated at 37°C for 1 h. After incubation, emission signals were collected at 665 nm using EnVision Multilable Reader (Perkin Elmer, United States).

### Animals

Male NC/Nga mice, 7-weeks-old, were purchased from Central Lab Animal Inc., South Korea. All mice were housed in specific pathogen-free conditions at the animal facility center of the College of Pharmacy at Seoul National University (Seoul, Korea), and were maintained at 24 – 26°C with a 12 h light and dark cycle. Animal experiments were conducted in accordance with protocols approved by the Institutional Animal Care and Use Committee of Seoul National University.

### Sensitization and Challenge

NC/Nga mice were classified into six groups (*n* = 6): (1) vehicle alone, acetone-olive oil (Sigma-Aldrich, St. Louis, MO, United States) in a ratio of 3:1 was used as a vehicle; (2) 1% of compound-**1** with vehicle alone; (3) 0.3% of compound-**2** with vehicle alone; (4) DNCB+vehicle; (5) DNCB + 1% of compound-**1** mixed with vehicle; and (6) DNCB + 0.3% of compound-**2** mixed with vehicle. DNCB (Sigma-Aldrich, St. Louis, MO, United States) was used to induce AD on the dorsal skin of the mice. Compound **1** and **2** were dissolved in 150 μl of vehicle and topically applied on the atopic tissue. Hair on the backs of the mice were removed using an electric clipper. Two days later, 150 μl of 1% DNCB was dissolved in the vehicle and applied on the dorsal skin twice with an interval period of 4 days. Afterward, 150 μl of 0.2% DNCB dissolved in the vehicle was applied to challenge the dorsal skin twice-a-week for 5 weeks. AD-induced mice were co-treated with either compound-**1** or compound-**2** once daily from day six onward until the end of the experiments.

### Evaluation of Skin Lesion

Dermatitis score was measured according to a previously established method, once-a-week for 5 weeks (Matsuda et al., 1997). Skin features of dermatitis, such as erythema/hemorrhage, edema, excoriation/erosion, and scaling/dryness were considered in the evaluation. Each symptom was scored as follows: 0 (none); 1 (mild); 2 (moderate); and 3 (severe). The sum of all the scores was defined as the dermatitis score of individual mice, which ranged between 0 and 12. In addition, the scratching behavior of the experimental animals was measured once-a-week for 5 weeks. Specifically, we measured the frequency with which the mice rubbed their dorsal skin, hind paws, nose, and ears for 10 min.

### Histopathology and Immunohistochemistry

Skin samples collected from the experimental mice were fixed by using 10% neutral buffered formalin, and embedded in paraffin. 4-μm thin tissue sections were prepared and stained with hematoxylin and eosin (H&E; Sigma-Aldrich, United States). To investigate the mast cell infiltration, sections were stained with toluidine blue (Sigma-Aldrich, United States). For immunohistochemical analysis, tissue sections were microwaved in sodium citrate buffer for 10 min to retrieve the antigen/epitope. After incubating with 0.1% triton X-100 containing phosphate buffered saline (PBS), sections were blocked with 10% FBS. Then, sections were incubated overnight with primary antibodies specific for IL-4, 5, 17, IFN-γ, and CD4, respectively (Santa Cruz Biotechnology, Santa Cruz, CA, United States). After treatment with 0.3% H_2_O_2_ in 0.1 M PBS for 15 min, sections were incubated with HRP-conjugated secondary antibodies specific for IL-4, 5, 17, IFN-γ, and CD4 for 1 h at room temperature. Diaminobenzidine was used to detect peroxidase activity, which was photographed using an Olympus AX70 light microscope (Tokyo, Japan).

### Analysis of Intracellular cAMP and TNF-α

The macrophage cell line, Raw 264.7 was maintained for 24 h in serum free DMEM medium. Then, cells were incubated with 30 nM of compound **1** and 15 nM of compound **2** for 1 h. After incubation, 1 μM of LPS (Sigma-Aldrich, United States) was added and incubated for 30 min at 37°C in a humidified 5% CO_2_ atmosphere. Finally, cells were lysed and supernatants were harvested. Intracellular cAMP levels were measured using the cAMP ELISA kit according to the manufacturer’s instruction (ADI-900-066; Enzo Life Sciences, United States). Similarly, TNFα level was assayed by ELISA analysis according to the manufacturer’s protocol (LEGEND MAX^TM^ Human TNF-α ELISA Kit, San Diego, CA, United States). Concentration is expressed in pg/mL.

### Measurement of Serum IgE, and Tissue IL-4, 5, 17 and IFN-γ

On the final day of the experimental scheme, blood samples were collected from the experimental animals. Serum was isolated and stored at -80°C along with protease inhibitor cocktail for later use (Thermo Fisher Scientific, United States). Serum IL-4, IL-5, IL-17, IFN-γ, and IgE levels were measured according to the manufacturer’s instructions (Biolegend, San Diego, CA, United States; IL-4-431101; IL-17-432501; IL-5-431204; IFN-γ- 430801; IgE-432401; TNF-α- 430901). Serum samples were diluted five-fold for the cytokine assay. 10,000-fold diluted serum samples were utilized for the IgE assay. Absorbance was measured immediately at 492 nm using a multiplate reader (Molecular Devices Spectramax M5, Sunnyvale, CA, United States).

### Statistical Analysis

The sample size of the experimental groups was determined based on the results obtained from the pilot study. Experimental animals were divided into three groups, with each group comprising at least six animals. Analysis of variance with a *post hoc* Tukey–Kramer test was performed to find the statistical significance between control and treated groups. Statistical significance was determined at *P* < 0.05.

## Results

### PDE Enzyme Inhibitor Assay

The PDE enzyme inhibitor assay was performed against PDE1A, PDE3B, PDE4A, PDE4B, PDE4C, PDE4D, and PDE7 to find the specificity and the inhibitory potential of compounds **1** and **2**. Observed results show that compound **2** exhibited high specificity against PDE4B compared to other PDE enzymes utilized in the study. The IC_50_ values of compound **2** against PDE1A, PDE3B, PDE4A, PDE4B, PDE4C, PDE4D, and PDE7 respectively, were 98 ± 8.45, 169 ± 3.78, 152 ± 1.33, 15 ± 0.4, 57 ± 4.87, 108 ± 3.78, and 250 ± 14.56 respectively. Compound **2** requires nearly sevenfold higher concentration to inhibit the PDE4D. Even though the compound **1** inhibited the PDE4B at a nano-molar concentration (31 ± 7.17 nM), its specificity is not exclusive to PDE4B alone (**Table 1**).

**Table 1:** The IC_50_ values of compounds **1** and **2** on various PDEs.

PDEs	IC_50_(nM)							
**Compounds**	**PDE1A**	**PDE3B**	**PDE4A4**	**PDE4B1**	**PDE4C1**	**PDE4D7**	**PDE7**

**1**	756 ± 58.98	800 ± 45.78	45 ± 3.54	31 ± 7.17	77 ± 8.8	220 ± 16.3	389 ± 26.3
**2**	98 ± 8.45	169 ± 3.78	152 ± 1.33	15 ± 0.4	57 ± 4.87	108 ± 3.78	250 ± 14.56

### PDE4 Inhibitors Alleviates AD-Like Symptoms in Mice

**FIGURE 1:**
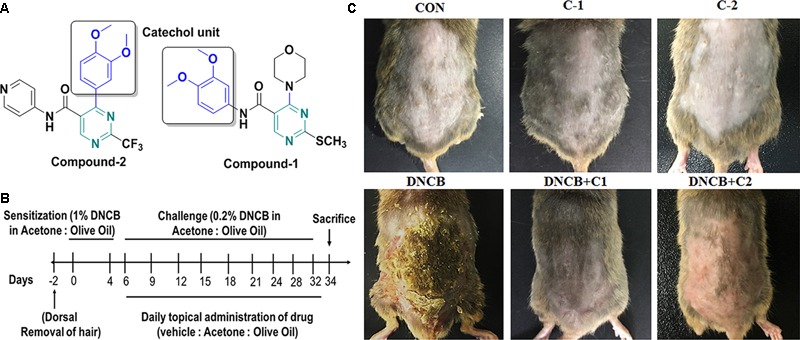
**(A)** Structure of compounds **1** and **2**. **(B)** Experimental scheme for the DNCB-induced AD model. On day 0 and day 4, the mice were sensitized with 150 μL of 1% DNCB or vehicle at their shaved back. From day 6 onwards 150 μL of 0.2% of DNCB or vehicle was applied to challenge the dorsal skin twice-a-week for 4 weeks. Atopic dermatitis-induced mice were co-treated with either compound-**1** or compound-**2** once daily from day six onwards until the end of the experiments. **(C)** Image represents the AD in experimental groups, which were taken before sacrifice. (CON- Control; C-1- Compound **1**; C-2- compound **2**; D+C1- DNCB+ Compound **1**; D+C2- DNCB+ Compound **2**).

**FIGURE 2:**
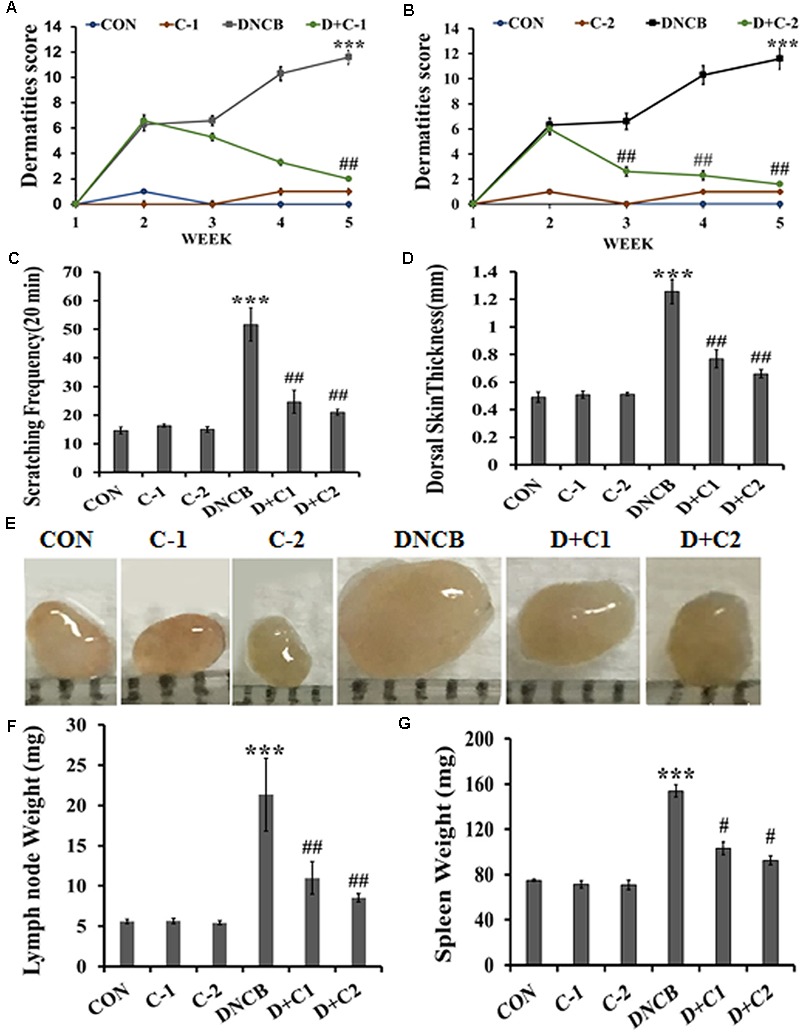
**(A,B)** Clinical skin severity was assessed through the epidermal skin score. Criteria for the skin score was described in materials and method. Epidermal skin score of compounds **1** and **2** treated AD induced mice were compared with control and DNCB-treated mice. **(C)** Scratching frequency in experimental animals. The frequency of scratching around the dorsal skin lesions and nose was counted during a 20-min period. Scratching frequency was measured once-a-week for 5 weeks. **(D)** The thickness of the dorsal skin of experimental mice. **(E)** Subiliac lymph node size. **(F)** Subiliac lymph node weight. **(G)** Spleen weight in experimental animals. (CON- Control; C-1, Compound **1**; C-2, compound **2**; D+C1, DNCB+ Compound **1**; D+C2, DNCB+ Compound **2**). Data represent the mean ± SEM (*n* = 6). ^∗∗∗^*p* < 0.001 vs. control; ^#^*p* < 0.5, ^##^*p* < 0.01 vs. DNCB.

**Figure 1A** shows the structure of compound **1** and **2**. The detailed scheme of synthesis is explained in supporting file. The scheme used to develop AD-like symptoms in NC/Nga mice is depicted in **Figure 1B**. All the experimental mice exhibited AD-like skin features after sensitization with 1% DNCB. AD-like symptoms such as itching, dry skin, erythema and hemorrhage were progressively accumulated in DNCB treated mice. Compound **1** and compound **2** treated AD induced mice showed reduced levels of erythema and hemorrhage and dry skin compared to DNCB alone induced mice. Compound **1** and compound **2** alone treated mice exhibited normal skin feature, no sign of allergy was observed (**Figure 1C**). The efficacy of the drug was analyzed through dermatitis score. Dermatitis scores were significantly very high and progressive in DNCB-alone treated mice as compared to the control group of mice (*p* < 0.001). A significant reduction (*p* < 0.01) in dermatitis score was observed after the third and second week, respectively, for the compound **1** and compound **2** treatments (**Figures 2A,B**). Scratching frequency was observed to monitor the itching behavior of the experimental mice. Compound **1** and **2** treatments significantly (*p* < 0.01) inhibited the DNCB –induced itching sensation in the experimental mice group (**Figure 2C**). At the end of the experiment, dorsal skin was peeled off and the skin thickness was measured. A pronounced increase in the dorsal skin thickness was observed (1.19 mm, *p* < 0.001) in DNCB-treated mice as compared to that of the control group (0.45 mm). Skin thickness of 0.78 mm and 0.64 mm were observed in compound **1** and **2** treated experimental mice (**Figure 2D**). During an inflammatory stress, lymph node size will increase. We observed a significant increase in the size and weight of the subiliac lymph node in DNCB-alone treated mice, as compared to the control mice. The size and weight of the lymph node are drastically reduced in both compounds **1-** and **2**-treated DNCB-induced mice, as compared to the DNCB-alone treated mice (**Figures 2E,F**). In addition, spleen weight was measured to assess the general health and immunological status of mice. It was found that topical application of DNCB markedly alters spleen weight, as compared to the vehicle alone-treated and drug-alone treated mice (*p* < 0.001). However, compound **2-** and compound **1**-treated atopic mice exhibited significantly reduced spleen weight (*p* < 0.5), as compared to that of DNCB-alone treated mice (**Figure 2G**).

### Histological Observation of the Skin

**FIGURE 3:**
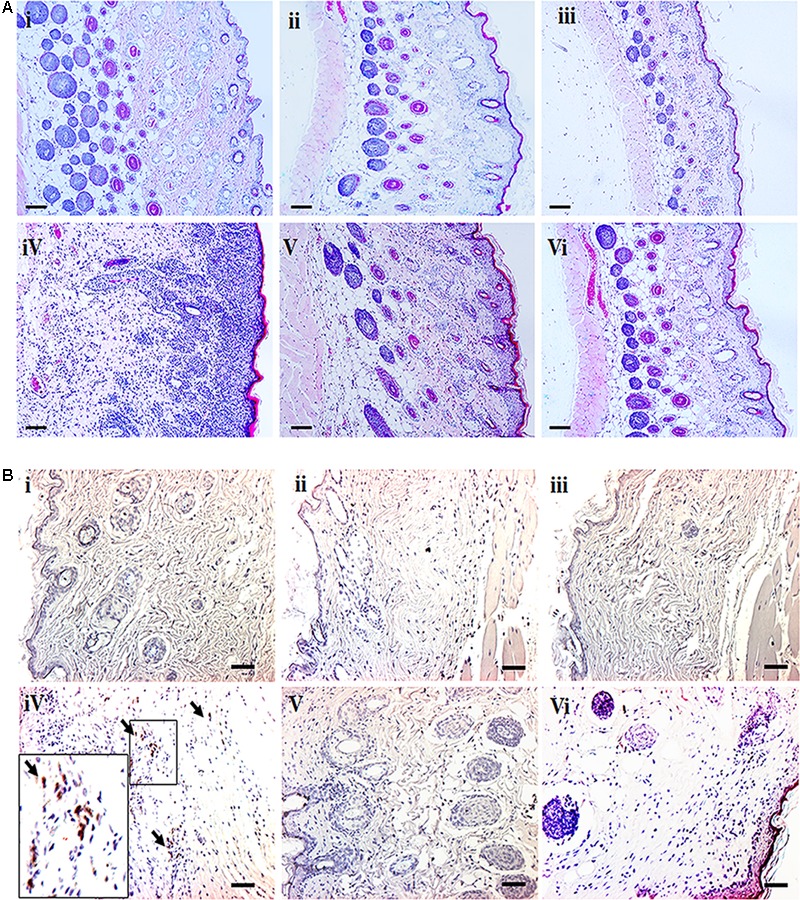
**(A)** H&E staining of dorsal skin of experimental animals. **(B)** Representative images depicting the immunohistochemical staining of CD4+ cells. The small black square indicates the location of CD4+ cells and is subsequently magnified in the lower left corner to observe the CD4+ cells. The arrows indicate CD4+ cells. (scale bar = 100 μm; magnification: x 100) (i- Control, ii- Compound **1** alone, iii- Compound **2** alone, iv- DNCB, v- DNCB+ Compound **1**, vi- DNCB+ Compound **2**).

**FIGURE 4:**
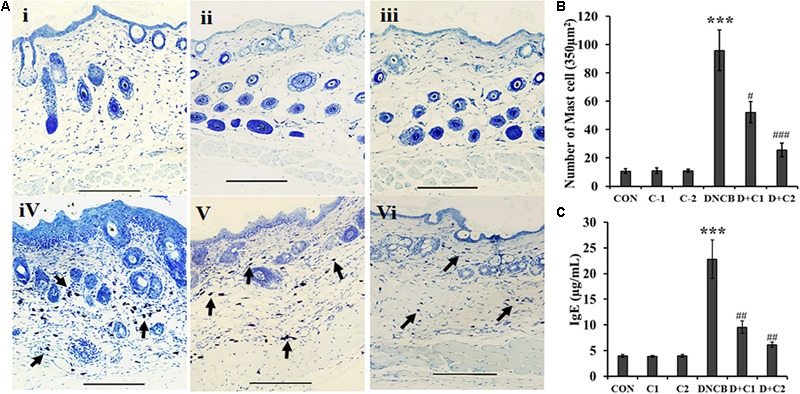
**(A)** Toluidine blue staining of mast cells. The arrows indicate mast cells. **(B)** Bar graph represent the number of mast cells counted per 350 μm^2^ at a magnification of x 400 in the epidermis region were counted **(C)** Serum IgE. (Scale bar = 100 μm; magnification: x 100) (i- Control, ii- Compound **1** alone, iii- Compound **2** alone, iv- DNCB, v- DNCB+ Compound **1**, vi- DNCB+ Compound **2**) Data represent the mean ± SEM (*n* = 3). ^∗∗∗^*p* < 0.001 vs. control; ^#^*p* < 0.5, ^##^*p* < 0.01 vs. DNCB.

H&E stained sections indicate dense infiltration of inflammatory cells into both epidermis and dermis region in DNCB–treated mice. In contrast, reduced levels of inflammatory cells were observed in compound **1** and **2** treated atopic mice. In addition, reduced epidermal thickening was observed in compound **1** and **2** treated atopic mice as compared to the DNCB-alone treated mice (**Figure 3A**). Immunohistochemical staining of CD4+ T cells revealed increased infiltration of CD4+ T cells in the DNCB-treated mice, as compared to the control, drug control, and treatment group of mice (**Figure 3B**). Similarly, increased levels of mast cell infiltration were observed in the epidermis region of DNCB-treated mice (97 ± 10.58, *p* ≤ 0.001), as compared to the control group (13 ± 2). However, significantly decreased mast cell infiltration was observed in compound **1** (56 ± 11, *p* ≤ 0.01) and compound **2** (29.41 ± 6, *p* ≤ 0.001) treated atopic mice (**Figures 4A,B**). IgE plays a key role in the degranulation of the mast cell. Serum IgE level in the DNCB-treated mice was significantly increased (sevenfold, *p* < 0.001), as compared to the normal and drug control mice. However, the DNCB-induced synthesis of IgE was significantly reduced in the compound **2** (*p* < 0.01) and compound **1** (*p* < 0.01) treated mice (**Figure 4C**).

### PDE4B Inhibitors Improves Intracellular cAMP Levels in Macrophage

**FIGURE 5:**
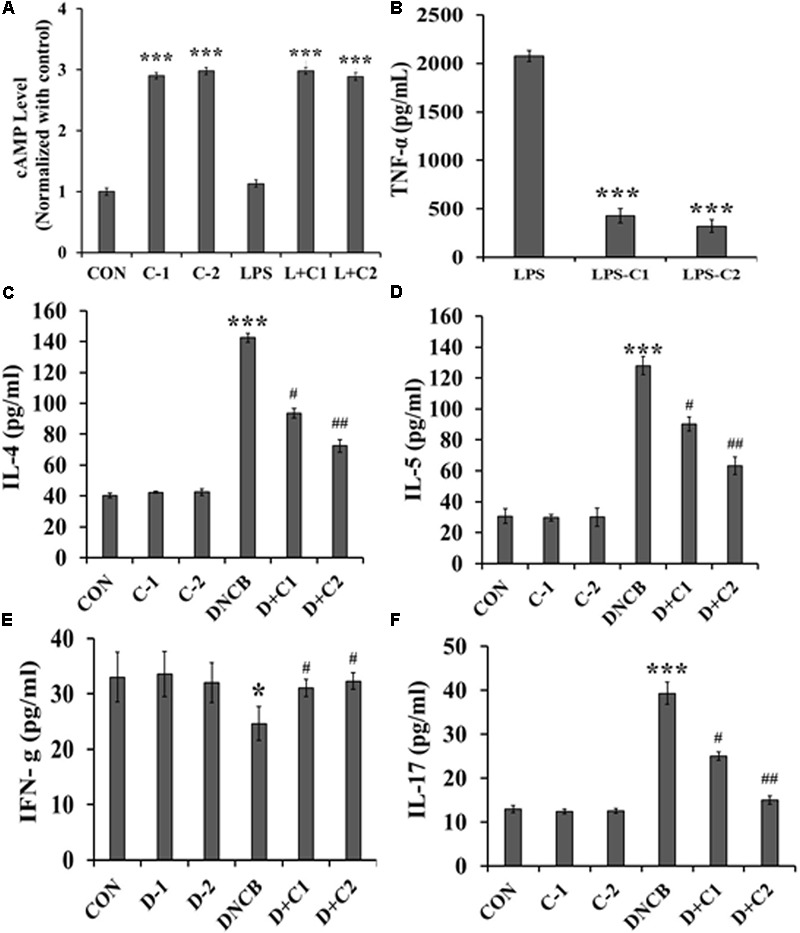
**(A)** cAMP levels in the cell lysate of compound **1** and compound **2** treated LPS stimulated Raw 264.7 cells. Cells were pretreated with 30 nM of compound **1** and 15nM of compound **2** for 1h, then stimulated with 1 μM of LPS for 30 min. cAMP levels were normalized with control. **(B)** The TNF-α level in LPS-treated Raw 264.7 cells of control, compound **1** and **2**. 30 nM of compound **1** and 15 nM of compound **2** treated cells were stimulated with 1 μM of LPS for 30 min. Serum cytokines level in experimental mice were quantified by ELISA: **(C)** Expression of serum IL-4. **(D)** Serum IL-5. **(E)** Serum IFN-γ. **(F)** Serum IL-17. Data represent the mean ± SEM (*n* = 6). ^∗^*p* < 0.5, ^∗∗∗^*p* < 0.001 vs. control; ^#^*p* < 0.5, ^##^*p* < 0.01 vs. DNCB.

The macrophage cell line, Raw 264.7 was pre-treated with compound **1** and **2** and then induced with lipopolysaccharide(LPS). The observed result in **Figure 5A** shows that cAMP levels were not significantly differed between control and LPS treated cells. However, cells treated with compound **1** and **2** significantly improved the intracellular levels of cAMP in macrophage cell line. Furthermore, much difference in the levels of cAMP was not observed between compounds **1** and **2** treated cells. It clearly implicates that PDE4B specific inhibition plays an important role in the maintenance of intracellular cAMP level in the macrophage. In a similar set of experiment, TNF-α levels were monitored. The PDE4 inhibitor treatment showed that both compound **1** and **2** effectively inhibited the LPS-induced TNF-α synthesis in macrophage cell culture (**Figure 5B**).

### Effect of Novel PDE4 Inhibitors on DNCB-Induced Inflammatory Mediators

**FIGURE 6:**
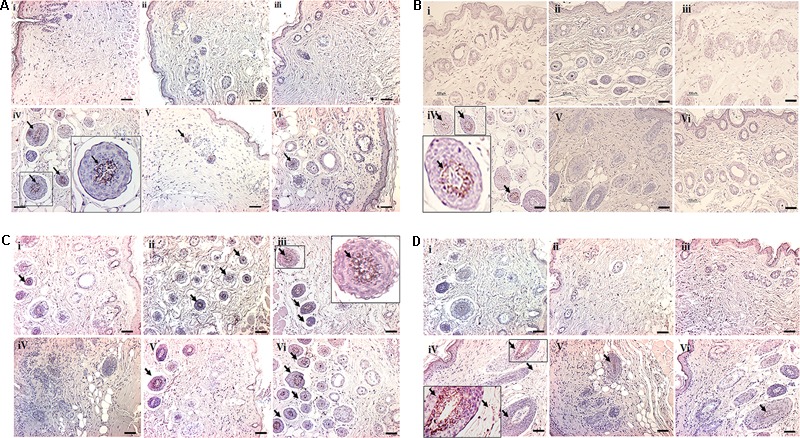
Immunohistochemical staining of cytokines deposited in the dorsal skin tissue of experimental animals. **(A)** Expression of IL-4. **(B)** Expression of IL-5. **(C)** Expression of IFN-γ. **(D)** Expression of IL-17. Scale bar indicates 100 μm (200X). The small black square indicates the location of antibody staining, which is subsequently magnified in the corners of the image. (i- Control, ii- Compound **1** alone, iii- Compound **2** alone, iv- DNCB, v- DNCB+**1**, vi- DNCB+**2**).

The levels of IL-4, 5, 17, and IFN-γ could reveal whether the immune response is Th1 cell-mediated or Th2 cell-mediated. As shown in **Figures 5C–E**, significantly increased levels of IL-4 (*p* < 0.001) and IL-5 (*p* < 0.001), but reduced IFN-γ levels (*p* < 0.5), were observed in the DNCB-alone treated mice, as compared to the control and drug control mice. However, the elevated levels of IL-4 and IL-5 were significantly reversed in the compound **2** (*p* < 0.01) and compound **1**- (*p* < 0.05) treated AD-induced mice. Similarly, the reduced IFN-γ level was significantly reversed in compound **2-** (*p* < 0.5) and compound **1-** (*p* < 0.5) treated AD-induced mice, as compared to the DNCB-alone treated mice. In addition, the level of IL-17 was observed to identify the involvement of Th17 + cells since Th17 + cells indicate the severity of AD. Increased levels of IL-17 (*p* < 0.01) were observed in DNCB-induced mice, as compared to the control mice. As expected, a reduced level of IL17 was observed in the compound **2-** (*p* < 0.01) and **1**- (*p* < 0.5) treated AD-induced mice, as compared to the DNCB-alone treated mice (**Figure 5F**). These results were further confirmed by immunohistochemistry, and the results are presented in **Figure 6**.

## Discussion

Phosphodiesterase inhibitors constitute attractive and promising anti-inflammatory agents. In the present study, DNCB was used to sensitize the skin of experimental mice which results in AD- like symptoms. It has been postulated that DNCB acts as a hapten, conjugate well with various skin proteins to elicit T- and B-cell mediated immunogenicity (Zhang et al., 2009). Sensitization of mice with DNCB generally stimulates unbearable itching sensation, which causes severe erythema, and edema at the affected site. In the present study, histopathological observation indicated that DNCB treatment stimulated the infiltration of immune cells toward affected area along with the severe erythema and edema. However, treatment of synthesized PDE4B inhibitor compound reversed the DNCB –induced symptoms. It clearly indicates the efficacy of the compound **2**.

Elevated levels of the intracellular cyclic AMP in the inflammatory cells favor the expression of anti-inflammatory mediators, such as IL-10, and suppression of pro-inflammatory mediators, such as IL-17, IL-4, and IL-5 (Hertz et al., 2009; Raker et al., 2016). For instance, increased levels of cAMP diminish the inflammatory potential of macrophages by suppressing the expression of cytokines (Hertz et al., 2009). The observed increased cAMP levels in compounds **1** and **2** treated macrophage clearly indicate that compounds **1** and **2** can improve the intracellular levels of cAMP in inflammatory cells. Interestingly, the result further indicates that PDE4B inhibition alone is sufficient to improve the intracellular level of cAMP in the inflammatory cells. Several lines of research reported an elevated PDE4B synthesis in T-lymphocytes, macrophages, and monocytes during allergic inflammation (Jin et al., 2005; Reyes-Irisarri et al., 2007). It has been reported that inhibition of PDE4D in the brain is the major reason for emesis. Hence, selective inhibition of PDE4B is highly beneficial since it could avoid the side effects such as emesis. *In vitro* enzyme inhibition assays observed in the present study clearly indicates that the compound **2** selectively inhibits the PDE4B. Being a selective inhibitor of PDE4B, compound **2** may avoid the side effects such as nausea and emesis. However, more clinical studies are warranted to confirm the anti-emetic potential of the compound.

Inflammatory mediators released by the activated T lymphocytes determine the sustenance and severity of AD. The development of memory T-lymphocytes requires cytokines, such as IL-4, IL-5, and IL-17 (Mannie et al., 2003). A number of clinical studies on AD patients revealed increased serum and tissue levels of IL-4 and IL-5, with a concomitant decrease in IFN-γ expression (Namkung et al., 2007; Vakirlis et al., 2011). It has also been reported that transgenic mice over expressing IL-4 and IL-5 were highly susceptible to AD (Elbe-Bürger et al., 2002; Lee and Flavell, 2004). In the present study, we also observed an elevated level of IL-4 and IL-5, and a decreased level of IFN-γ in DNCB-induced animals, as compared to that of vehicle and drug control animals. These abnormal cytokine levels were reversed in the compounds **1-** and **2**-treated AD-induced mice. However, the basal level of IFN-γ was observed in compounds **1-** and **2**-treated AD-induced mice similar to that of control mice. It should be noted that, in the present study, DNCB treatment promoted the infiltration of the activated CD4+ cell population at the AD-affected skin tissue in the experimental NC/Nga mice. It has been well documented that activated CD4+ cell elicits Th2-responsive cytokines, such as IL-4 and IL-5 (Mikhalkevich et al., 2006). The observed reduced levels of CD4+ cells in the skin of compound **1-** and **2**-treated AD-induced mice could be a reason for the reduced IL-4 and IL-5 levels in these experimental animals, as compared to the DNCB-alone treated mice. It seems that synthesized PDE4B inhibitors effectively raised cAMP levels in the inflamed skin. IL-17 is an important pro-inflammatory cytokine reported in the AD, which is secreted by Th17 cells, a subset of CD4+ cells (Di Cesare et al., 2008). It has been reported in the clinical samples of patients with a severe AD that pronounced levels of IL-17 are correlated with the severity of AD (Chiricozzi et al., 2014). The observed results of the present study indicate that compound **1** and **2** significantly suppressed the expression of DNCB-induced IL-17, which shows the possible involvement of cAMP-mediated signaling in the regulation of Th17 cells. Apart from this, the result observed in the present study shows that PDE4B inhibition suppresses the TNF-α levels in macrophage cell line Raw 264.7. It is well known that TNF-α activates the pro-inflammatory transcription activator, NF-κB. Interestingly, in a transgenic PDE4B-/-, PDE4A-/-, and PDE4D-/-null macrophage studies, it was reported that PDE4B alone, but not PDE4A/PDE4D, was involved in the suppression of LPS-induced secretion of TNF-α (Jin et al., 2005).

Pruritus affects the quality of life of AD patients. During inflammation, degranulation of mast cells in the epidermis and dermis region considerably stimulate the pruritus by releasing pruritogen-like histamine and leukotriene B4 (Weller et al., 2005). IgE plays a crucial role in eliciting type I hypersensitivity reactions by stimulating the degranulation of mast cells (Darsow and Ring, 2008). The binding of IgE to Fc𝜀 RI receptor of mast cells enables the mast cells to recognize allergen, which in turn provokes the degranulation of mast cells. Activated Cd4+ cells secreting cytokines, such as Il-4, IL-5, and IL-13 stimulate the class switch to IgE in B-cells (Tangye et al., 2002; Geha et al., 2003). In the present study, histopathological observation showed increased levels of mast cells in the epidermis region of AD-induced mice. However, compound **1** and **2** significantly suppressed the DNCB-induced infiltration of these inflammatory cells. Incomparable to the infiltrated mast cell, nearly 10-fold increase of serum IgE is observed in the DNCB treated mice, but compounds **1** and **2** significantly suppressed (*p* < 0.001) the DNCB-induced total serum IgE levels. The observed results in the present study indicate that catecholopyrimidine core containing compound **1** and compound **2** effectively inhibit the AD. However, compound **2** yield better results compared to compound **1**.

## Conclusion

In Summary, the novel PDE4 inhibitor, compound **2**, exhibits antipruritic and anti-inflammatory effects against DNCB-induced AD in NC/Nga mice with low IC_50_ value (15 ± 0.4 nM) against PDE4B. The catecholopyrimidine core of compound **2** mimics the cAMP, this property enhances the probability of binding interactions at the catalytic domain of the enzyme. The results obtained in this study demonstrated that compound **2** could improve the symptoms of the AD through inhibiting the release of Th2 cytokines IL-4, 5 and 17 as well as suppressing the activation of mast cells via suppressing the synthesis of IgE. These results strongly suggest that compound **2** might constitute a particularly efficient PDE4B inhibitor molecule for the treatment of allergic diseases.

## Author Contributions

BP designed and synthesized the PDE inhibitors. PA performed the biological experiments. Both BP and PA wrote the manuscript. JMS designed the study and revised the manuscript.

## Conflict of Interest Statement

The authors declare that the research was conducted in the absence of any commercial or financial relationships that could be construed as a potential conflict of interest.
