# Supplementary material for: A Novel Catecholopyrimidine Based Small Molecule PDE4B Inhibitor Suppresses Inflammatory Cytokines in Atopic Mice
Source: Front Pharmacol. 2018 May 11;9:485. doi: 10.3389/fphar.2018.00485 (PMC5958743; doi:10.3389/fphar.2018.00485)
Supplement: Supplementary file 1 [file Data_Sheet_1.docx]

**Supplementary Material**

**A Novel Catecholopyrimidine based Small Molecule PDE4B Inhibitor Suppresses Inflammatory Cytokines in Atopic Mice**

Baskaran Purushothaman^†^, Parthasarathy Arumugam^†^, and Joon Myong Song*

College of Pharmacy, Seoul National University, Seoul 151-742, South Korea

**Experimental section**

The compounds **1** and **2** were synthesized in good quantity for the animal studies by our previously reported procedure (Purushothaman et al., 2017).

**Scheme 1.** Synthesis of compound-1:

**Synthetic procedure for compound-1**

1) Ethyl 4-chloro-2-(methylthio)pyrimidine-5-carboxylate **(4)**

To a solution of diethyl (ethoxy methylene) malonate **1**, (1 g, 4.62 mmol) and S-methylisothiourea hemisulfate salt **2** (0.9650 g, 6.93 mmol) in anhydrous EtOH (10 mL) was added sodium ethoxide (0.47 g, 6.93 mmol). Then the reaction mixture was refluxed at 80⁰C for 8 h under N_2_ atmosphere. TLC confirmed the completion of the reaction. Then the reaction mixture was cooled to room temperature and evaporated the excess ethanol by rotavapor and poured into ice water to get solid. The solid was filtered and washed with DI water and hexane, dried under vacuum to give the ethyl 2-(methylthio)-6-oxo-1,6-dihydropyrimidine-5-carboxylate (**3)** as a pale yellow solid (0.5 g, 50%), which was used without further purification for next step. The intermediate **3** (0.5 g) was slowly added to phosphoryl (V) oxychloride (5 ml) and the reaction mixture was heated to 100⁰C for 3h. After cooling the reaction mixture to room temperature, slowly poured into crushed ice. Slowly stirred to get the solid, then filtered the solid and purified by column chromatography on silica gel (5% EtOAc in Hexane) to afford intermediate **4** as an off-white solid. Yield: 0.5 g, 92.59%. ESIMS: 233 (M+1).

2) Ethyl 2-(methylthio)-4-moropholinopyrimidine-5-carboxylate **(5)**

To a mixture of Ethyl 4-chloro-2-(methylthio)pyrimidine-5-carboxylate (0.5 g, 2.14 mmol) and N,N-diisopropylethylamine (0.767 mL, 4.29 mmol) in 2-propanol (8 mL) was added morpholine (0.224 mL, 2.57 mmol). The mixture was stirred at room temperature for 3 h and was then concentrated in vacuo. The resulting residue was purified by column chromatography (40% EA/Hex) to give Ethyl 2-(methylthio)-4-moropholinopyrimidine-5-carboxylate as a white solid (0.6 g, 100%). ESIMS: 284 (M+1).

3) 2-(methylthio)-4-morpholinopyrimidine-5-carboxylic acid **(6)**

To a solution of Ethyl 2-(methylthio)-4-moropholinopyrimidine-5-carboxylate (0.6 g, 2.12 mmol) in EtOH (10 mL) was added 1 N NaOH aqueous solution (2 mL) and the reaction mixture was stirred at room temperature for overnight. The organic solvent was evaporated and 1 N HCl aqueous solution (10 mL) was added to the residue. The resulting precipitate was collected by filtration and dried under high vacuum pump to give the title compound (0.4 g, Yield: 74%) as a white solid. ESIMS: 256 (M+1).

4) N-(3,4-dimethoxyphenyl)-2-(methylthio)-4-morpholinopyrimidine-5-carboxamide **(7)**

To a solution of 2-(methylthio)-4-morpholinopyrimidine-5-carboxylic acid (**6)** (1 mmol) in anhydrous DMF (5 ml) were added N-(3-dimethylaminopropyl)-N-ethylcarbodiimide hydrochloride (EDCI.HCl) (1.2 mmol), Hunig’s base (2.5 mmol) and Hydroxybenzotriazole (HOBt) (1.2 mmol). Stirred the reaction mixture for 30 min to dissolve and activate the acid starting material at 0°C then to room temperature for 1 h. Then 3,4-dimehtoxyaniline (1 mmol) was added and stirred the reaction mixture for overnight at room temperature under N_2_ atmosphere. Then the reaction mixture was slowly poured into DI water and extracted with DCM (50 ml). The organic layer was washed with sat.NaHCO_3_ and then dried over anhydrous sodium sulfate. The crude product was purified by column chromatography using silica gel as stationary phase.

Appearance: off-white Solid. Yield: 450 mg, 73.77%. IR: 3262, 1639, 1519, 1399, 1247, 1165, 1126 cm^-1^. ^1^H NMR (400 MHz, DMSO-*d*_6_) δ 10.33 (s, 1H), 8.21 (s, 1H), 7.35 (d, *J* = 2.4 Hz, 1H), 7.20 (dd, *J* = 8.7, 2.4 Hz, 1H), 6.92 (d, *J* = 8.7 Hz, 1H), 3.73 (d, *J* = 2.5 Hz, 6H), 3.63 (dd, *J* = 5.5, 3.4 Hz, 4H), 3.55 (dd, *J* = 5.4, 3.5 Hz, 4H), 2.48 (s, 3H).^13^C NMR (500 MHz, CDCl_3_) δ 173.31, 163.81, 160.93, 157.86, 149.30, 146.24, 131.25, 111.75, 111.46, 111.34, 104.50, 66.55, 56.15, 56.01, 48.64, 14.18. (ESI+): C_18_H_22_N_4_O_4_S Calcd: 390.4580, found: 391.1436 (M+1).

**Scheme 2.** Synthesis of compound-2:

**Synthetic procedure for compound-2**

1) ethyl-4-chloro-2-(trifluoromethyl)pyrimidine-5-carboxylate (**11**)

The intermediate **11** was synthesized by following the same as Ethyl 4-chloro-2-(methylthio)pyrimidine-5-carboxylate (**4**). ESIMS: 255 (M+1).

2) Ethyl 4-(3,4-dimethoxyphenyl)-2-(trifluoromethyl)pyrimidine-5-carboxylate **(12)**

A solution of P(t-Bu)_3_ (50 mg, 0.23 mmol) and Tris(dibenzylideneacetone)dipalladium(0) (Pd_2_(dba)_3_) (0.1 g, 0.117 mmol), in 10 ml of THF, stirred at room temperature for 30 min, the dark red heterogeneous solution Pd(P(t-Bu)_3_)_2_ formed. Then KF (0.478 g, 8.24 mmol), and 3,4-dimethoxyphenylboronicacid (0.428 g, 2.35 mmol) in dry THF (10 ml) was added. Finally ethyl-4-chloro-2-(trifluoromethyl)pyrimidine-5-carboxylate (0.3 g, 1.17 mmol), was added under N_2_ atmosphere. The reaction mixture was heated to 55⁰C for 17 h. TLC showed the completion of the reaction. Then the mixture was cooled and concentrated under vacuo to get crude residue, which was purified by column chromatography over silica gel (Ethylacetate/ Hexane, 1:3) to afford title product as an off-white solid in 95.23% yield. ESIMS: 357 (M+1).

3) 4-(3,4-dimethoxyphenyl)-2-(trifluoromethyl)pyrimidine-5-carboxylic acid **(13)**

Ethyl 4-(3,4-dimethoxyphenyl)-2-(trifluoromethyl)pyrimidine-5-carboxylate (0.4 g, 1.4 mmol) in ethanol (15 mL) was added 1 N NaOH aqueous solution (2 ml). Then the reaction mixture was stirred at room temperature for overnight. TLC confirmed the completion of the reaction. The organic solvent was evaporated and 1 N HCl aqueous solution (1 ml) was added to the residue. The resulting precipitate was collected by filtration and dried under high vacuum pump to give the title compound as a white solid, Yield: 0.35 g, 97.22%. ESIMS: 329 (M+1).

4) 4-(3,4-dimethoxyphenyl)-N-(pyridin-4-yl)-2-(trifluoromethyl)pyrimidine-5-carboxamide **(14)**

To a solution of 4-(3,4-dimethoxyphenyl)-2-(trifluoromethyl)pyrimidine-5-carboxylic acid (1 mmol) in 5 ml of dry DCM were added oxalyl chloride (2 mmol) and anhydrous DMF (catalytic amount) at 0⁰C. The reaction mixture was stirred at 0⁰C for 30 min then to room temperature for 1hr. The reaction mixture was concentrated under N_2_ atmosphere in vacuo to give crude 4-(3,4-dimethoxyphenyl)-2-(trifluoromethyl)pyrimidine-5-carbonyl chloride as a pale yellow solid. This compound was used for next reaction without further purification. To this crude material 5 ml of dry DCM and triethylamine (2.5 mmol) was added, followed by pyridine-4-amine (1 mmol) at 0 ⁰C. The reaction mixture was stirred at 0 ⁰C for 15 minutes, then slowly to room temperature for 1 hour. The reaction mixture was concentrated in vacuo. The resulting residue was purified by column chromatography using silica gel as a stationary phase.

Appearance: pale yellow solid. Yield: 0.4 g, 93%. TLC (CH_2_Cl_2_: CH_3_OH, 9:0.5 v/v) R_f_ = 0.4; IR: 1687, 1591, 1266, 1146, 1024, 814 cm^-1^; ^1^H NMR (500 MHz, CDCl_3_) δ 9.06 (s, 1H), 8.43 – 8.38 (m, 2H), 8.07 (s, 1H), 7.48 (dd, J = 8.4, 2.2 Hz, 1H), 7.42 (d, J = 2.2 Hz, 1H), 7.37 – 7.32 (m, 2H), 6.93 (d, J = 8.4 Hz, 1H), 3.90 (s, 3H), 3.82 (s, 3H); ^13^C NMR (500 MHz, CDCl_3_) δ 164.61, 163.04, 158.74, 152.61, 150.83, 149.58, 144.06, 128.39, 126.61, 123.33, 120.30, 113.60, 111.76, 111.33, 56.12, 56.03, 31.57, 22.63, 14.10; (ESI+) m/z C_19_H_15_F_3_N_4_O_3_ Calcd: 404.3492, found: 405.1403 (M+1).

**^1^H NMR for Compound-1**


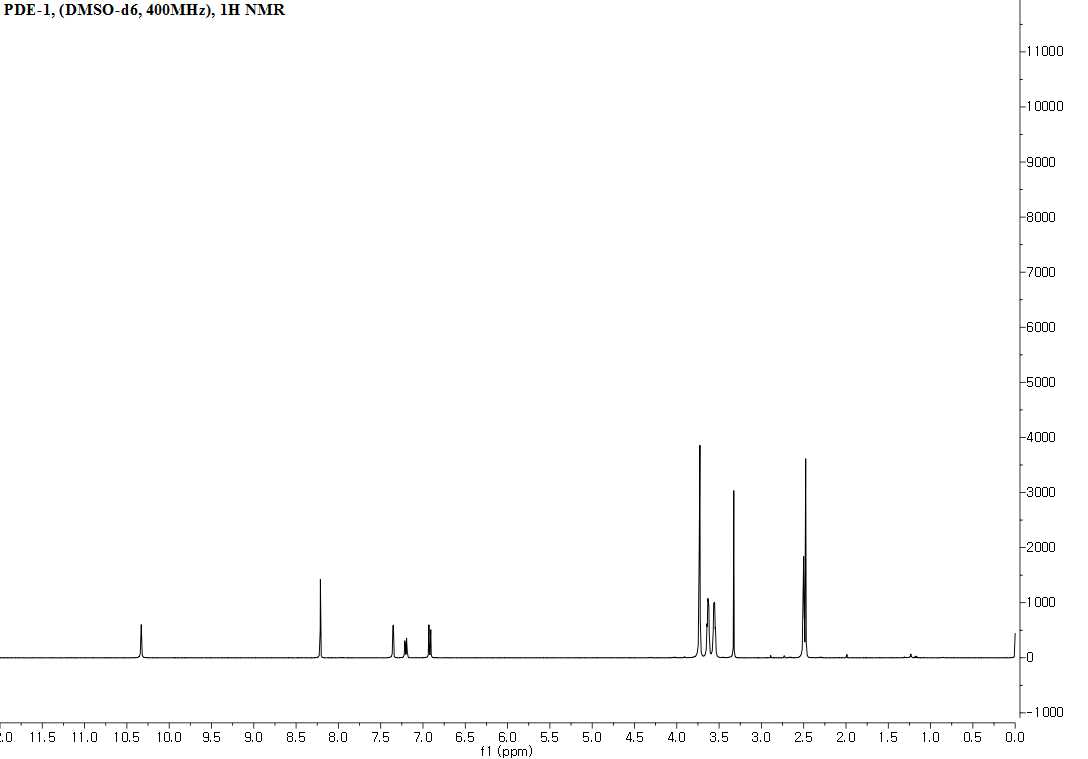


**^13^C NMR for Compound-1**


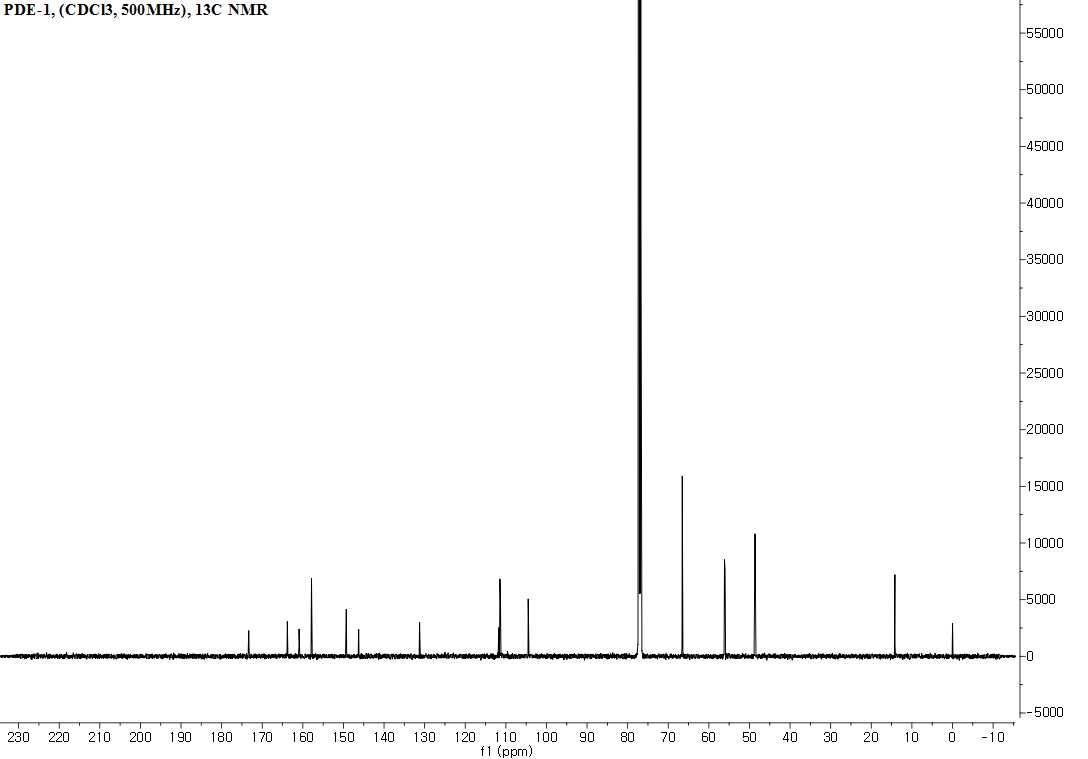


**^1^H NMR for Compound-2**


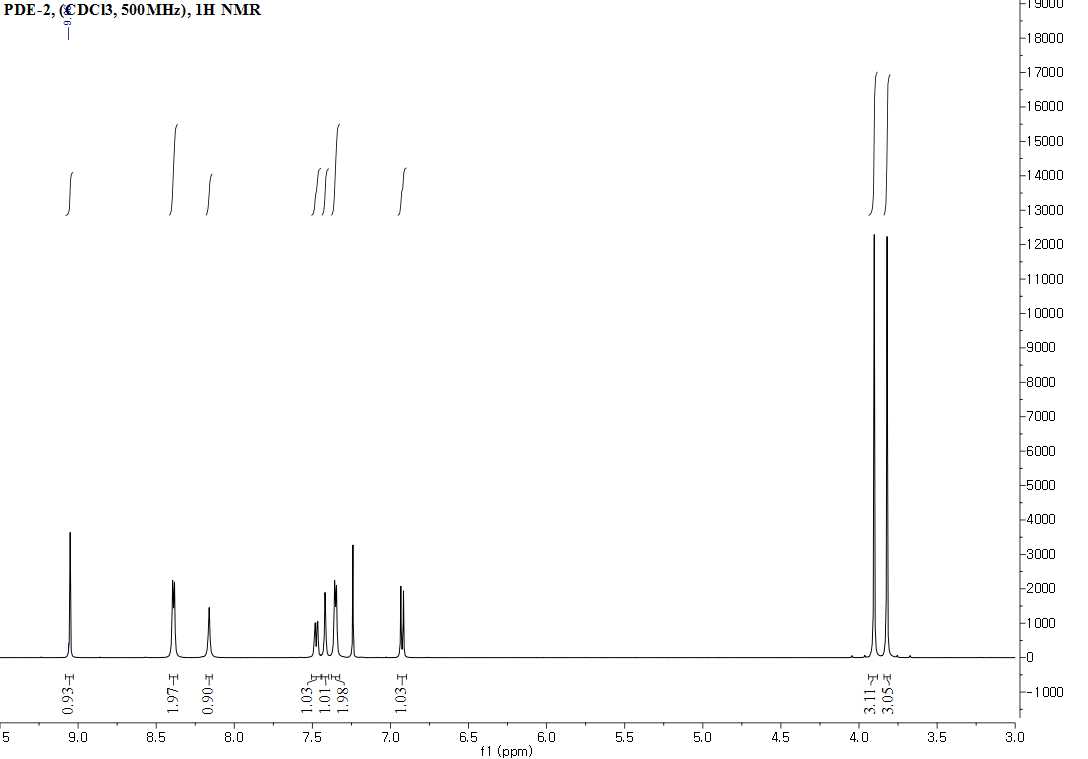


**^13^C NMR for Compound-2**


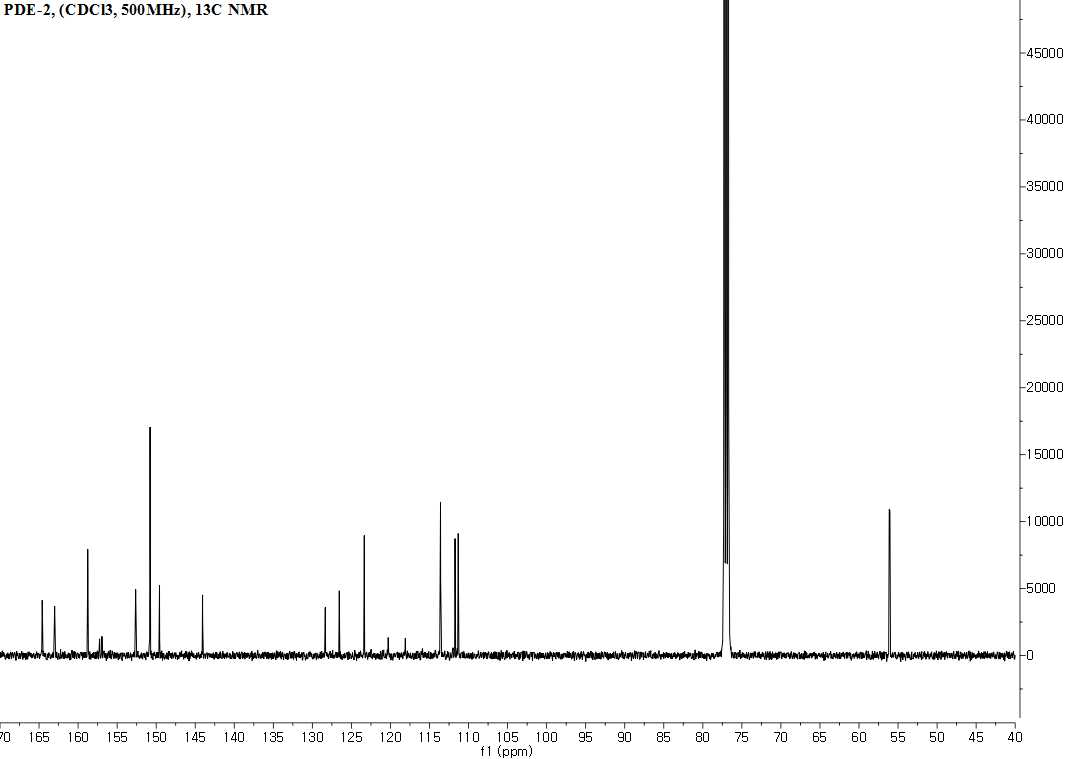


**Reference:**

Purushothaman, B., Arumugam, P., Kulsi, G., and Song, J. M. (2017). Design, synthesis, and biological evaluation of novel catecholopyrimidine based PDE4 inhibitor for the treatment of atopic dermatitis. *Eur. J. Med. Chem.* 145, 673–690. doi: 10.1016/j.ejmech.2017.12.069.
